# Development of a Molecularly Imprinted Pencil Graphite Electrode for the Voltammetric Detection of Hg^2+^ Ions

**DOI:** 10.1021/acsomega.5c07433

**Published:** 2026-01-23

**Authors:** Mehmet Karagözlü, Dina El Miari, Mariam Moghazi, Süleyman Aşır, Ilgım Göktürk, Fatma Yılmaz, Adil Denizli, Deniz Türkmen

**Affiliations:** † Research Center of Science, Technology and Engineering (BILTEM), 52988 Near East University, Mersin 10, Nicosia 99138, Turkey; ‡ Department of Food Engineering, Faculty of Agriculture, Near East University, Mersin 10, Nicosia 99138, Turkey; § Department of Biomedical Engineering, Faculty of Engineering, University of Kragujevac, Kragujevac 34000, Serbia; ∥ Department of Biomedical Engineering, Faculty of Engineering, Near East University, Mersin 10, Nicosia 99138, Turkey; ⊥ Department of Chemistry, 37515 Hacettepe University, Ankara 06800, Turkey; # Department of Chemistry and Chemical Processing Technologies, 52942 Bolu Abant Izzet Baysal University, Bolu 14900, Turkey

## Abstract

The ability to detect trace amounts of mercury has become critical due to its possible severe toxic effects on both humans and the environment when exposed to unsafe amounts. This study focuses on developing a novel mercury detection technique using differential pulse anodic stripping voltammetry (DPASV) with a pencil graphite electrode modified with a molecularly imprinted polymer (MIP-PGE). The amino acid-based N-methacryloyl-(L)-cysteine methyl ester (MAC) monomer was used for the imprinting process of Hg^2+^ ions. Fourier transform infrared spectroscopy equipped with an attenuated total reflection (FTIR-ATR), contact angle (CA), and scanning electron microscopy (SEM) was used to characterize the electrochemical sensors. The obtained results revealed that the voltammetric detection method using MIP-PGE was able to accurately detect Hg^2+^ in both aqueous solutions and real samples for different concentrations, with an acceptable linear relationship. This novel sensor demonstrated low detection and quantification limits of 0.188 nM and 0.570 nM, respectively. The produced sensor offers rapid, simple, and cost-effective detection of Hg^2+^ ions.

## Graphical abstract

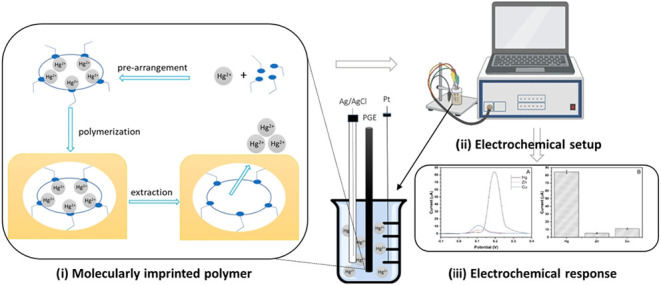

## 1 Introduction

Mercury (Hg) is considered one of the most dangerous chemical elements on Earth due to its severe effects on human health and the environment following long-term exposure. Hg is a significant environmental concern due to its high toxicity and widespread presence. However, the toxicity of Hg strongly depends on its chemical form. In aqueous environments, Hg primarily exists in two forms: inorganic mercury (Hg^2+^) and organic mercury, such as methylmercury (CH_3_Hg). CH_3_Hg, produced by aquatic bacteria from Hg^2+^, is extremely dangerous due to its easy penetration and significant bioaccumulation, making it more hazardous than Hg^2+^ ions.^1^ In the case of Hg, the organic species are much more toxic than the inorganic ones. The most important of them is CH_3_Hg, whose lethal dose ranges from 20 to 60 mg kg^–1^ for a 70 kg person. CH_3_Hg, effectively absorbed in the gastrointestinal tract, then crosses the blood–brain and placental barriers, causing irreversible damage to the central nervous system. The increased toxicity of organic mercury species is due to their lipophilic properties and slow elimination rate from organisms, leading to bioaccumulation.^2^ So, Hg^2+^ ion exposure, even in small concentrations, causes poisoning, and severe exposure harms different organs within the body, such as the brain and kidneys. As a neurotoxin, mercury can induce symptoms similar to those of a neurological disorder.^3^ Environmentally speaking, through human influence, mercury can be removed from its deposits and transported to the environment, eventually being methylated into CH_3_Hg by the activity of microorganisms. This organic form of Hg can be accumulated and moved through the food chain, causing severe harm to animals and humans.^4,5^ The principal pathway by which people encounter CH_3_Hg is through the eating of fish, which can lead to mercury poisoning. The World Health Organization (WHO) has established a permissible limit for long-term inhalation exposure to mercury vapor as elemental mercury, at 0.2 μg/m^3^. WHO also suggests 2 μg per kilogram of body weight as a tolerable daily intake of total mercury and 2 μg/L for drinking water.^6,7^ As a result, the increasing levels of CH_3_Hg and Hg^2+^ in our environment, driven by both natural and human-generated mercury emissions, are a pressing concern for the ecosystem and public health. So, identifying the permissible maximum concentration of total Hg is crucial for ensuring safety and protecting public health. Several methods have been developed to detect mercury in a variety of matrices as required by different standards. These methods include spectrophotometry,^8^ atomic absorption spectrometry (AAS),^9^ inductively coupled plasma mass spectrometry (ICP-MS),^10^ and inductively coupled plasma optical emission spectrometry (ICP-OES).^11^ Traditional methods of detecting mercury are currently faced with several problems, including being less sensitive while registering high detection limits.^12^ Also, these techniques require complex sample preparation and are not suitable for real-time monitoring of mercury species in aquatic environments and live cells.

Electrochemical sensors based on molecularly imprinted polymers (MIPs) and ion-imprinted polymers (IIPs) have been widely used in clinical diagnosis, food safety, and environmental monitoring.^13^ The detection of heavy metals like mercury ions (Hg^2+^) has been greatly improved by MIP electrodes.^14^ The materials imitate the biological macromolecules’ molecular recognition mechanism, e.g., substrate–enzyme or antigen–antibody interactions.^15^ MIPs are formed by arranging functional monomers around a template molecule and then linking them together. The monomers are self-assembled around the template molecule through interactions among functional groups in the template and the monomers.

Initially, MIPs are synthesized through a cross-linking process. Subsequently, the template molecule is either partially or entirely removed from the polymer network. This removal creates specific recognition sites within the polymer that are complementary in size and shape to the original template molecule. These meticulously formed pores enable the material to selectively recognize and bind to target molecules that structurally resemble the original template.^15,16^ MIP-based sensors offer superior performance compared to traditional sensors, exhibiting enhanced sensitivity, selectivity, environmental robustness, and cost-effectiveness.^17–19^ When integrated with electrochemical detection, these MIPs enable the fabrication of sensors that clearly distinguish between different types of target molecules with exceptional sensitivity and specificity.^17,20^ MIPs possess selectivity by binding solely with the target molecule, while the electrochemical detection method guarantees sensitivity by detecting negligible fluctuations in the electrical attributes because of the attachment of the target molecule to the MIPs.^20–22^

While some simultaneous sensors rely primarily on ‘peak potential discrimination’ to separate signals of different heavy metals, the MIP-based sensor proposed in this work utilizes a ‘lock-and-key’ molecular recognition mechanism. This structural and chemical affinity provides robust selectivity, especially in complex matrices where the peak potentials of interfering ions might shift or overlap. Furthermore, unlike traditional electrodes, such as glassy carbon (GCE) or gold electrodes used in many simultaneous detection studies, the use of PGE as a transducer substrate offers exceptional cost-effectiveness and commercial ubiquity without compromising sensitivity.

In this study, a novel amino acid-based electrochemical biosensor was developed by Hg^2+^-imprinted poly­(hydroxyethyl methacrylate-N-methacryloyl-(L)-cysteine methyl ester) polymer (MIP-PGE) for Hg^2+^ detection. For this purpose, NIP-PGE (nonimprinted poly­(hydroxyethyl methacrylate-N-methacryloyl-(L)-cysteine methyl ester)) polymer, as well as MIP-PGEs that have different monomer-to-target ratios, were synthesized. Characterization of the synthesized PGEs was carried out using scanning electron microscopy (SEM) to investigate surface morphology as well as contact angle (CA) measurements. Additionally, Fourier transform infrared spectroscopy equipped with an attenuated total reflection (FTIR-ATR) module was employed for detailed polymer analysis. The electrochemical analysis was performed using differential pulse anodic stripping voltammetry (DPASV) for mercury detection. Different parameters, including pH, deposition potential, and deposition time, were investigated to determine the optimal conditions for the analysis. The analytical performance of the produced biosensor for different concentrations of Hg^2+^ ions was evaluated for both the standard solution and the real sample. In addition, selectivity and imprinting efficiency studies were conducted.

## 2 Materials and Methods

### 2.1 Materials and Reagents

Monomers (2-hydroxyethyl methacrylate (HEMA), ethylene glycol dimethacrylate (EGDMA), and l-methacryloyl chloride, initiator (azoisobisbutyronitrile (AIBN)), and ethylendiaminetetraacetic acid (EDTA) were used in the synthesis of polymers. Acetate buffers (ABS) (0.5 M) were made at pH 4.5 and 5.5 using sodium acetate and acetic acid. The pH 1.5, 2.5, and 3.5 buffers were prepared using sodium dihydrogen phosphate and phosphoric acid. As a desorbing agent, a 0.05 M EDTA solution was prepared. All of these substances were obtained from Merck (Darmstadt, Germany). Deionized water (18.2 MΩ cm), used in the preparation of all aqueous solutions, was supplied by Purelab Ultra Analytic (ELGA Lab Water, Runcorn, UK). Mercury­(II) sulfate, copper­(II) nitrate hemipentahydrate, lead­(II) nitrate, cadmium nitrate tetrahydrate, and zinc nitrate hexahydrate were supplied by Sigma-Aldrich. Two millimeter pencil graphite leads were bought from the local stationery store. Artificial plasma solutions were obtained from ClinCheck (Recipe, Munich, Germany).

### 2.2 Apparatus

The polymeric layers on the three MIP-PGEs and NIP-PGE were analyzed by using Fourier transform infrared spectroscopy with an attenuated total reflection module (FTIR-ATR, Thermo Fisher Scientific, Nicolet iS10, Waltham, MA, USA) within the wavenumber range of 400–4000 cm^–1^. Then, scanning electron microscopy and contact angle measurements of the selected electrode (MIP-B) were carried out compared to bare PGE and NIP-PGE. Following the deposition of a thin gold–palladium alloy coating, the surface morphology of bare PGE, MIP-PGE, and NIP-PGE was examined using scanning electron microscopy (SEM, JSM-6400, JEOL, Akishima, Tokyo, Japan). Surface characterization of the bare, MIP-PGE, and NIP-PGE was also carried out by performing ten contact angle measurements at different surface locations using the sessile drop method with a Kruss DSA100 contact angle device (Hamburg, Germany). Hg^2+^-imprinted MIP-PGEs and NIP-PGE were characterized by using Fourier transform infrared spectroscopy.

The AUTOLAB PGSTAT204 potentiostat/galvanostat (Metrohm, Utrecht, The Netherlands) was used with the NOVA 2.1.2 software to perform DPASV. In electrochemical analysis, a three-electrode cell setup was used for the experiment. Ag/AgCl with 3 M KCl was used as a reference electrode. Platinum wire was used as the counter electrode. MIP-PGE and NIP-PGE were used as the working electrodes. For the deposition, duration between 10 and 150 s, voltage between −0.3 and −0.9 V and an interval time of +0.01 s were used. The parameters of DPASV measurement for start potential, stop potential, step height, and pulse amplitude were set as −0.5 V, +0.5 V, 0.005 mV, and 0.025 V, respectively. All electrochemical experiments were conducted at room temperature in triplicate to ensure reproducibility and statistical significance.

### 2.3 Preparation of MIP-PGEs

*N*-methacryloyl-(L)-cysteine methyl ester (MAC), serving as both a comonomer and a metal-chelating ligand, was synthesized according to previously reported methods.^23^ In summary, l-cycteine hydrochloride (5.0 g) and hydroquinone (0.2 g) were dissolved in 100 mL of dichloromethane, and the solution was cooled to 0 °C. Triethylamine (13.0 g) was subsequently added, followed by the gradual addition of methacryloyl chloride (4.0 mL) under a nitrogen atmosphere. The reaction mixture was stirred at room temperature for 2 h. To remove any unreacted methacryloyl chloride, the mixture was extracted with a 10% NaOH solution. The aqueous phase was then evaporated by using a rotary evaporator, and the resulting MAC product was dissolved in ethanol for further use. Using the synthesized MAC, three different Hg^2+^-imprinted MIP-PGEs were prepared with MAC:Hg^2+^ ratios of 2:1, 1:1, and 1:2 (mmol/mmol), designated as MIP-A, MIP-B, and MIP-C, respectively (Table 1). For each formulation, the respective MAC-Hg^2+^ precomplex was mixed with EGDMA (2 mmol) and HEMA (0.4 mmol) for 1 h, after which AIBN (5 mg) was added as the initiator. After the PGEs were immersed in the monomer mixtures, polymerization was completed by exposing the PGEs under UV light (356 nm) for 1 h. Using the same procedure, NIP-PGE was prepared by excluding Hg^2+^ ions.

**Table 1:** Synthesis Parameters for the PGEs

**electrode**	**ratio (monomer: Hg** ^**2+**^ **)**	**monomer**	**template**	**cross-linker**	**initiator**	**solvent**
MIP-A	2:1	MAC	Hg^2+^	EGDMA	AIBN	EtOH
MIP-B	1:1	MAC	Hg^2+^	EGDMA	AIBN	EtOH
MIP-C	1:2	MAC	Hg^2+^	EGDMA	AIBN	EtOH
NIP		MAC		EGDMA	AIBN	EtOH

Additionally, a 0.05 M EDTA solution was prepared and used as a desorbing agent to rinse all working electrodes throughout the study. The purpose of this procedure was to effectively remove any residual Hg^2+^ ions bound to the electrode surfaces, thereby enhancing the analytical accuracy and improving the electrode affinity. For each step, the electrodes were immersed in the EDTA solution for approximately 1 min, followed by a 1 min rinse in distilled water, after which they were ready for subsequent measurements.

## 3 Results and Discussion

### 3.1 Characterization Studies

Figure 1 presents the Fourier transform infrared (FTIR) spectrum obtained using the attenuated total reflectance (ATR) method for different monomer:template ratios of Hg­(II)-MAC complex ((a) 1:1, (b) 1:2, (c) 2:1 mmol:mmol) for MIP-PGEs and NIP-PGE (Figure 1d). A characteristic absorbance peak at 945.12 cm^–1^ can be attributed to the – SH stretching vibration of MAC monomer in the NIP-PGE structure. The peak observed in the −SH region for NIP-PGE disappears as the amount of template Hg^2+^ increases in the MIP structure, indicating that the −SH group coordinates with Hg^2+^ ions in the polymer form. The sulfhydryl groups can donate the lone pair of electrons to the empty orbital of the metal ions alone. Also, the carbonyl band observed at 1718.58 cm^–1^ shifts to the higher wavenumber values upon coordination to the Hg^2+^ ions.

**Figure 1:**
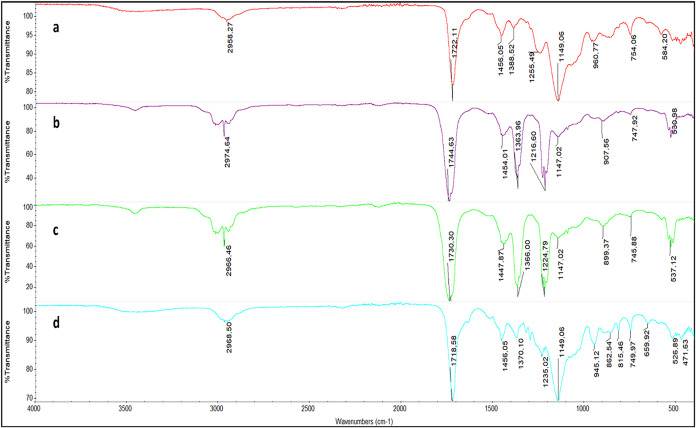
FTIR-ATR spectrum of the PGEs: (a) MIP-B, (b) MIP-C, (c) MIP-A, and (d) NIP-PGE.

Figure 2a–c displays the SEM images of the PGEs, captured at identical magnification, illustrating their surface morphology. Molecular imprinting generally results in the formation of a polymeric layer surrounding the template molecule, producing surface cavities that contribute to a rougher texture. Correspondingly, contact angle (CA) images of the PGEs are presented in Figure 2d–f. The measured CA values for NIP-PGE, MIP-PGE, and bare PGE were 116.7° ± 0.14, 113.3° ± 0.28, and 109.7° ± 0.28, respectively. The decreased CA value observed for the MIP-PGE suggests enhanced surface hydrophilicity, which is attributed to the coordination of Hg^2+^ ions with MAC during the molecular imprinting process.

**Figure 2:**
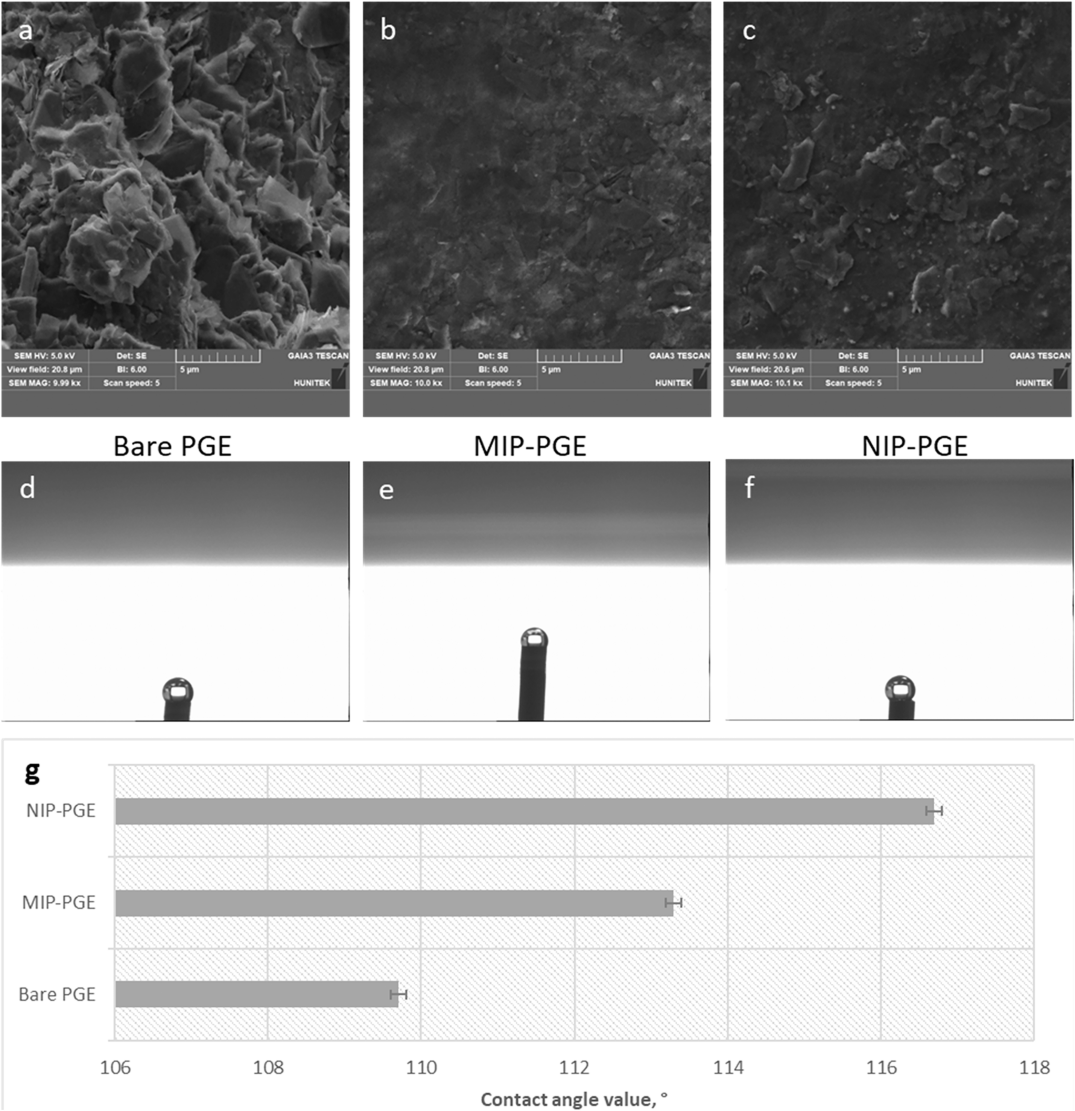
SEM and contact angle image measurements of the PGEs. SEM images of PGEs: (a) bare PGE, (b) MIP-PGE, and (c) NIP-PGE. Contact angle images of PGEs: (d) bare PGE, (e) MIP-PGE, (f) NIP-PGE, and (g) contact angle values of PGEs.

### 3.2 Optimization of the MIP-PGE Sensor

Figure 3A,B represents the voltammograms for 100 μM Hg^2+^ solutions with various pH values and the peak current vs pH graph, respectively. Different buffer solutions were tested for optimization of Hg^2+^ ions with pH values of 1.5, 2.5, 3.5, 4.5, and 5.5 by using DPASV, respectively. It was found that the 0.5 M ABS buffer at a pH of 4.5 showed the highest peak when using a concentration of 100 μM Hg^2+^, and it was therefore selected as the supporting electrolyte. The optimal pH of 4.5 for Hg^2+^ detection with the MIP-PGE sensor is likely due to the protonation state (protonated or deprotonated) of the N-methacryloyl-(L)-cysteine methyl ester (MAC) monomer. At this pH, the sulfhydryl (–SH) groups on the MAC are optimally deprotonated, allowing for effective chelation and strong coordination with Hg^2+^ ions. This specific deprotonation maximizes the binding affinity and stability of the Hg^2+^-MAC complex within the polymer network, leading to enhanced detection. Since deposition was used to reduce Hg^2+^ ions first to elemental mercury Hg, optimization of this deposition process was required to obtain the best responses. The optimization of deposition potential was done for the applied potential of −0.3, −0.4, −0.5, −0.6, −0.7, −0.8, and −0.9 V at deposition time of 75s (Figure 3C,D), and then the optimization of deposition time was done at different deposition timings of 10, 30, 50, 75, 100, 125, and 150 s at an applied potential of −0.7 V (Figure 3E,F), respectively. It was found that an applied potential of −0.7 V for 75 s yielded the best response, as it was sufficiently short to be efficient while producing the highest peak.

**Figure 3:**
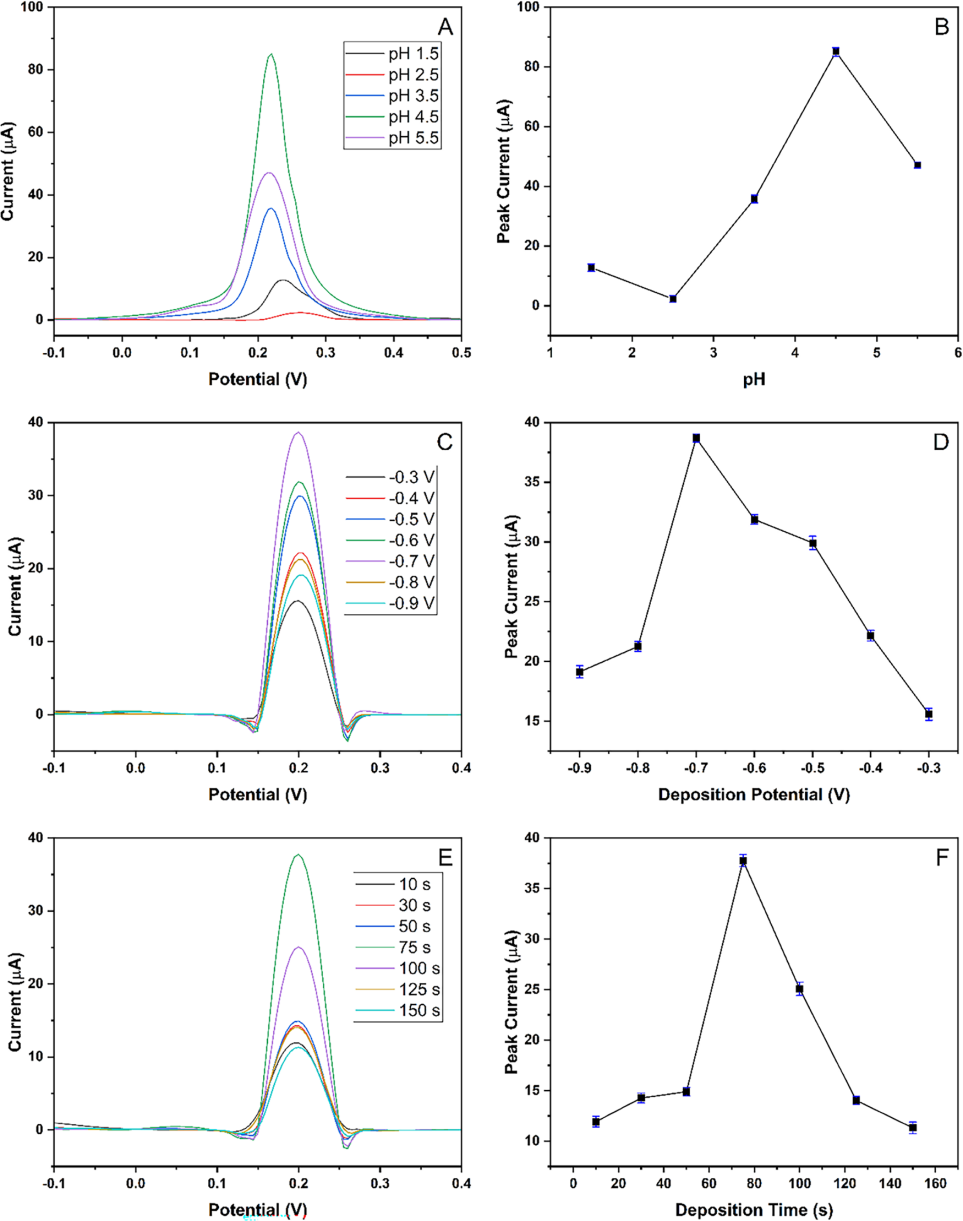
Optimization studies. (A) DP voltammograms for 100 μM Hg^2+^ solutions with various pH values. (B) Peak current vs pH graph of the DPASV responses. (C) DP voltammograms recorded after applying different deposition potentials (deposition time 75s) for a solution of 50 μM Hg^2+^. (D) peak current vs deposition potential graph of DPASV responses. (E) DP voltammograms recorded after different deposition times for a 50 μM Hg^2+^ solution. (F) peak current vs deposition time graph for DPASV responses.

Moreover, three different MIP-PGEs, with the monomer-to-template (mercury) ratios of 2:1, 1:1, and 1:2 as MIP-A, MIP-B, and MIP-C, respectively, were used to determine the selection of the MIP-PGE electrode. As represented in Figure 4, the MIP-B, named as MIP-PGE in the paper, with a 1:1 ratio, gave the highest peak response and was selected as the working electrode for the electrochemical measurements.

**Figure 4:**
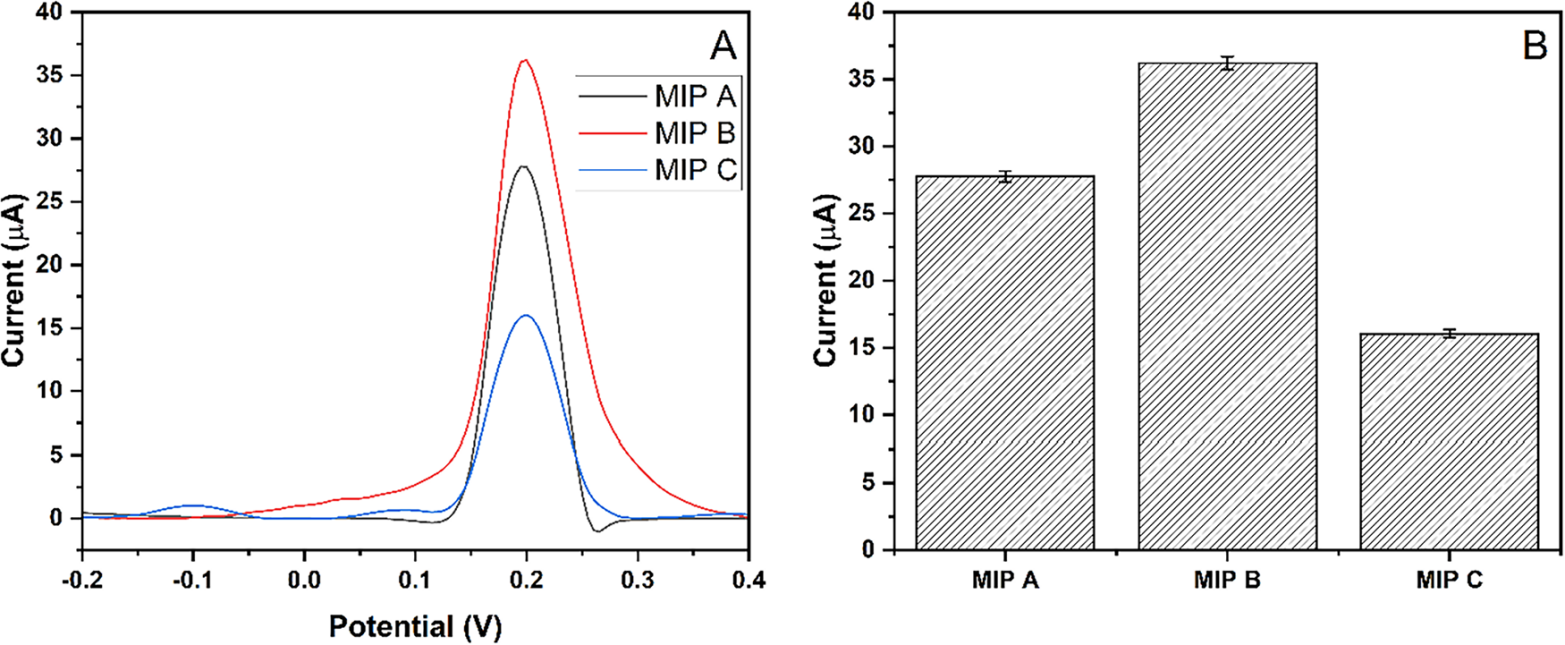
Selection of MIP-PGE electrodes with different monomer-to-Hg^2+^ ratios. (A) DPASV response of MIP-PGEs for 50 μM Hg^2+^. (B) Column representation of the MIP-PGEs responses with error bars.

### 3.3 Analytical Performance of the MIP-PGE

Differential pulse anodic stripping voltammetry was used for electrochemical analysis after the chronoamperometry. Different concentrations of Hg^2+^ solutions in 0.5 M ABS (pH 4.5), including 1, 10, 15, 25, 50, 75, and 100 μM, were prepared and analyzed. As represented in Figure 5, an acceptable linear relationship was obtained with peak currents as the concentrations of Hg^2+^ ions increased from 1 to 100 μM. As shown in Figure 5B, the linear regression equation for Hg^2+^ ions is calibrated as *I =* 0.8452 C – 2.0064 with a correlation coefficient (*R* ^2^) of 0.9946. To further validate the sensor performance in the critical 150 nM to 1 μM range, complementary experiments were conducted. DPASV measurements were performed for Hg^2+^ concentrations from 0.2 to 0.75 μM. The voltammograms and the corresponding calibration curve are provided in the Supporting Information (Figure S1). The linear relationship for this range (*I* = 1.2611 C + 1.1089 and *R* ^2^ = 0.9631) confirms the reliable operation of MIP-PGE across a wider concentration spectrum, which is particularly relevant for environmental and health monitoring standards.

**Figure 5:**
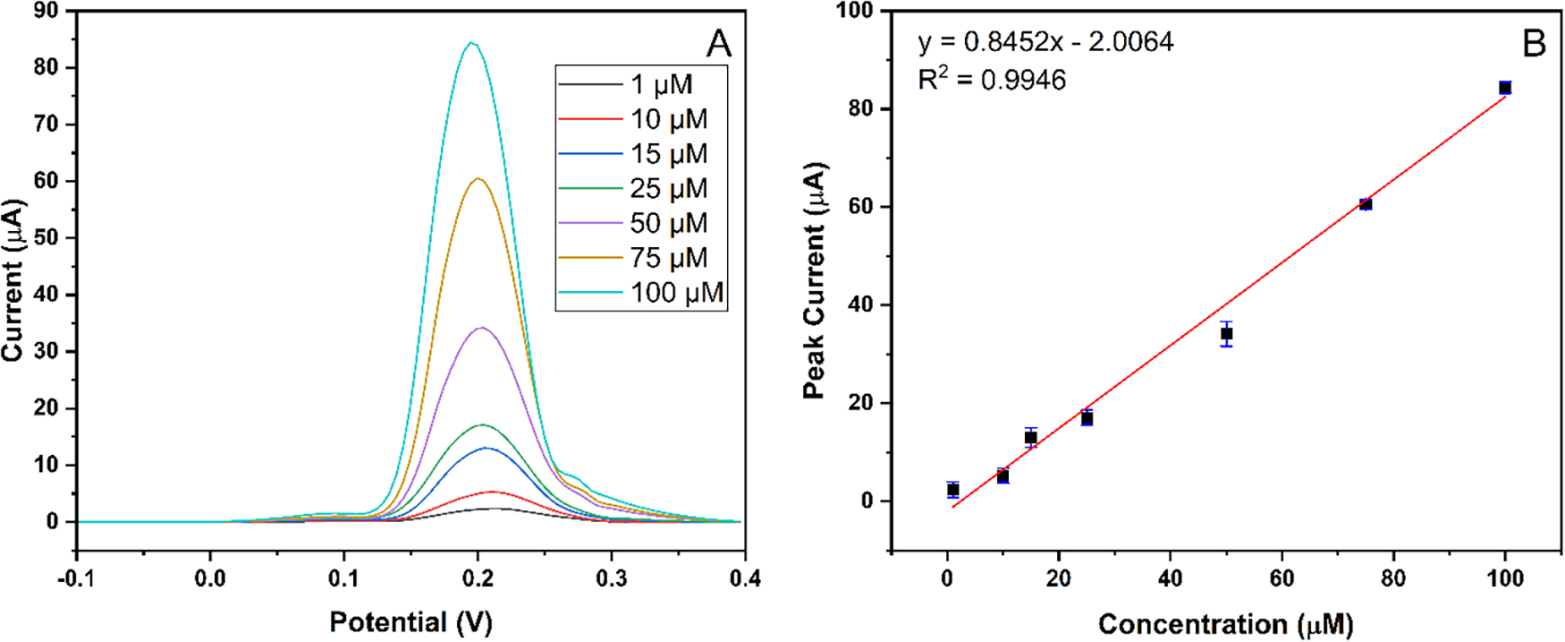
Response of MIP-PGE at various μM concentrations. (A) Voltammogram of Hg^2+^ by DPASV via MIP-PGE electrode. (B) Calibration graph of Hg^2+^ in concentrations ranging from 1 to 100 μM.

In addition, the developed MIP-PGE electrode’s sensitivity in detecting Hg^2+^ ions at the nanomolar level was examined. In that manner, 25, 50, 75, 100, and 150 nM concentrations of Hg^2+^ in 0.5 M ABS (pH 4.5) were prepared and detected. Figure 6 reveals that with increasing concentration, the peak current responses increase linearly. The linear regression equation for Hg^2+^ ions is calibrated as *I =* 0.00665 *C +* 0.14697 with an *R* ^2^ of 0.9904, as represented in Figure 6B. Limit of detection (LOD) and limit of qualification (LOQ) were calculated by following equations:

1 $$\mathrm{LOD}=\frac{3.3\;\mathrm{SD}}{m}$$

2 $$\mathrm{LOQ}=\frac{10\;\mathrm{SD}}{m}$$

where SD and *m* represent the standard deviation and the slope of the calibration curve, respectively. The LOD and LOQ values of the produced electrode were 0.188 and 0.570 nM, respectively. As a result, the relatively higher regression coefficient (*R* ^2^: 0.9946) and the higher slope value reported for the high Hg^2+^ concentration range (1–100 μM) indicate that the driving force for the adsorption and mass transfer mechanism probably stems from the concentration difference. However, also for the lower Hg^2+^ concentration (25–150 nM) range, the remarkably higher regression coefficient (*R* ^2^: 0.9904) indicates the linearity of the MIP-PGE responses for Hg^2+^ detection with a low detection limit of 0.188 nM.

**Figure 6:**
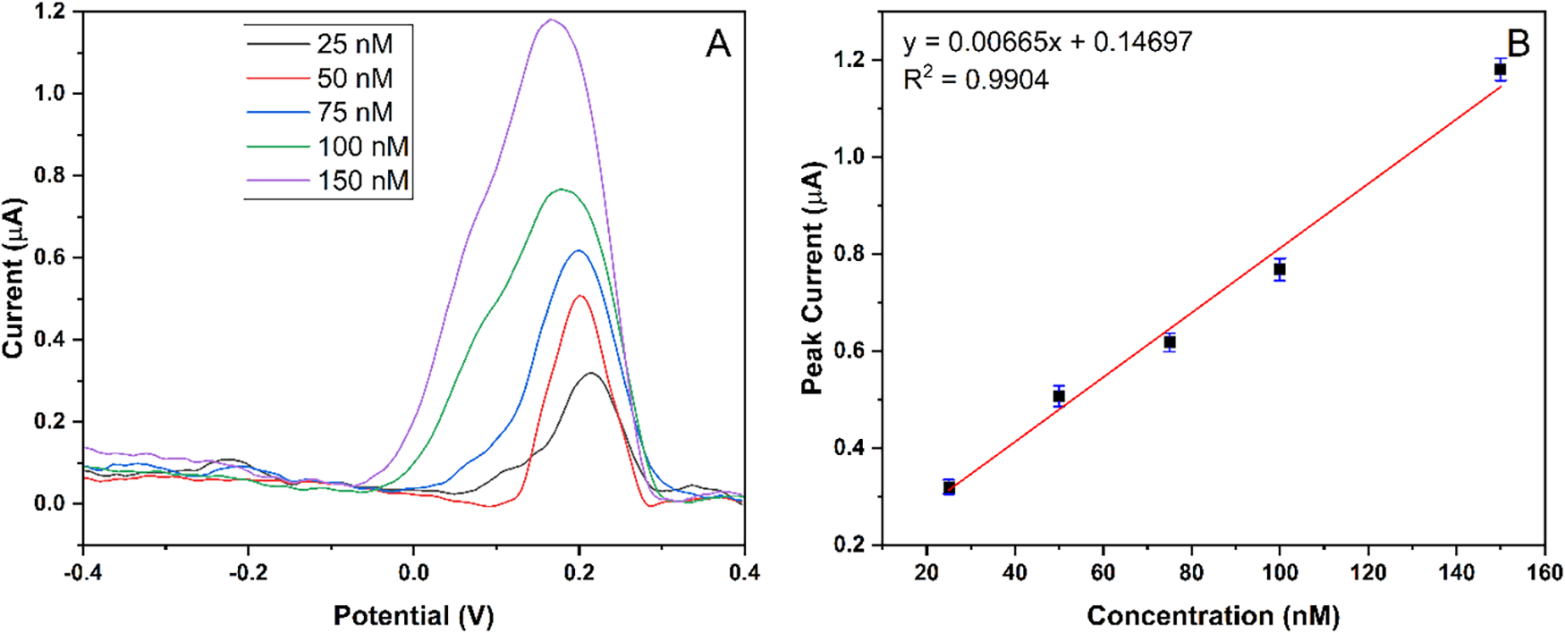
Response of MIP-PGE in various nM concentrations. (A) Voltammogram of Hg^2+^ by DPASV via MIP-PGE electrode. (B) Calibration graph of Hg^2+^ in concentrations ranging from 25 to 150 nM.

### 3.4 Estimation of Selectivity

There is growing interest in using sensors as biological recognition elements by integrating them with molecularly imprinted polymers. This innovative process enables template molecules to precisely guide the arrangement of structural components using a cross-linking agent, resulting in enhanced specificity and sensitivity. The template is then removed, leaving behind cavities that match the template molecules in shape, size, and steric configuration. The molecular imprinting method enables the production of synthetic polymers that possess unique molecular recognition capabilities for different templates. Molecularly imprinted polymers exhibit strong chemical and physical structures that offer remarkable mechanical strength and excellent resistance to high pressure, extreme temperatures, acids, and alkalis. Their easy synthesis facilitates long-term performance, reusability, and recyclability, rendering them highly valuable for a wide range of applications.^24^

The selectivity of the electrochemical sensor was tested using MIP-PGE with individual competing ions Cu^2+^, Pb^2+^, Cd^2+^, and Zn^2+^, as represented in Figure 7. The selectivity coefficients (*k*) for both competing ions were calculated by taking the response ratios of Hg^2+^ to Cu^2+^, Pb^2+^, Cd^2+^, and Zn^2+^, as shown in Table 2. In addition, the MIP-PGE response to a mixture of Hg^2+^, Cu^2+^, Pb^2+^, Cd^2+^, and Zn^2+^ in the same solution is given in Figure S2.

**Figure 7:**
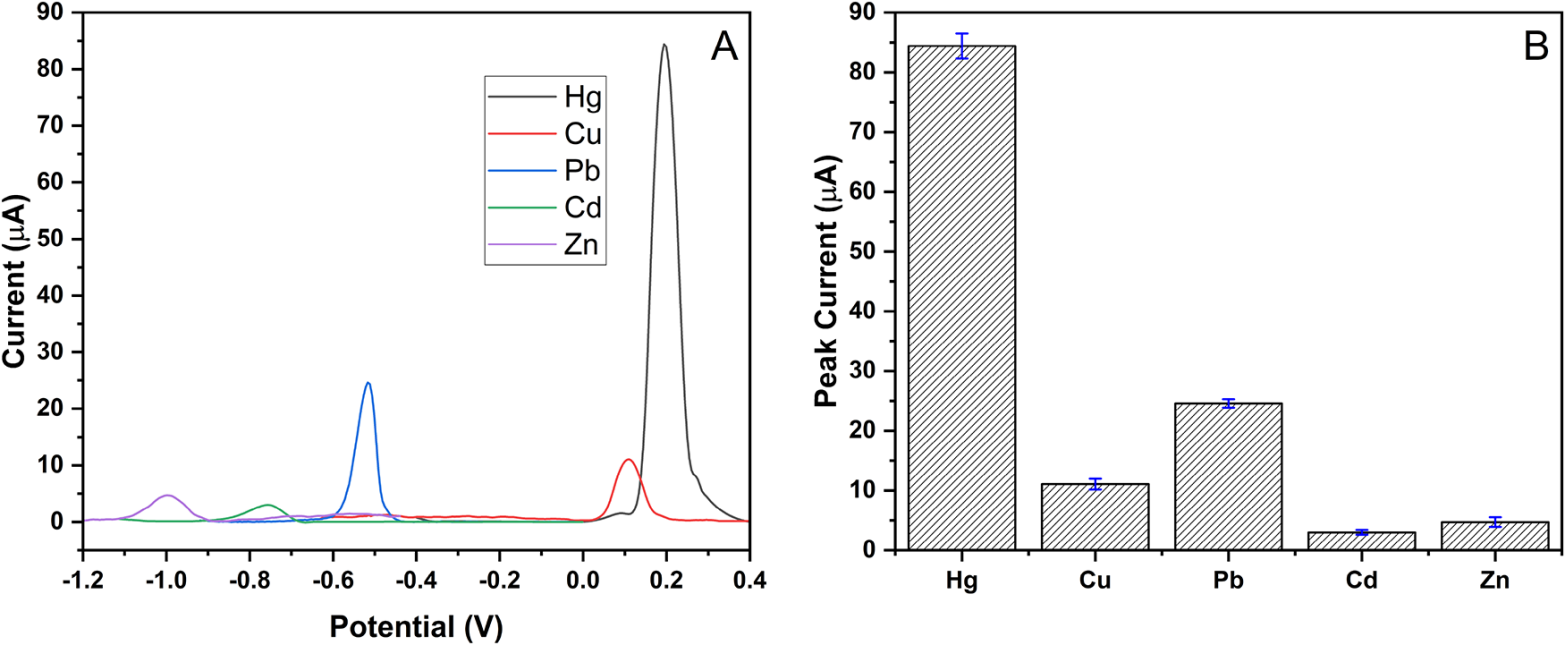
Selectivity study. (A) MIP-PGE response to Hg^2+^ (100 μM), Cu^2+^ (100 μM), Pb^2+^ (100 μM), Cd^2+^ (100 μM), and Zn^2+^ (100 μM) competitors. (B) Column representation of the MIP-PGE responses with error bars.

**Table 2:** Peak Heights Obtained in Selectivity Studies and Selectivity Coefficients

	**MIP-PGE**	
	**peak height (μA)**	***k***
Hg^2+^	84.40	
Cu^2+^	11.08	7.62
Pb^2+^	24.59	3.43
Cd^2+^	3.02	27.95
Zn^2+^	4.71	17.92

The results demonstrate that MIP-PGE is 7.62, 3.43, 27.95, and 17.92 times selective for Hg^2+^ compared to Cu^2+^, Pb^2+^, Cd^2+^, and Zn^2+^, respectively. This improved selectivity has been attributed to the imprinting process, where templates are shaped cavities within polymers, causing them to bind specifically to Hg^2+^ and thereby increasing their selectivity and affinity. From the results, it can be concluded that Hg^2+^ was detected selectively without the need for complex processes such as ligand immobilization or the use of spacer arms. To assess the sensor’s performance in a complex matrix where intermetallic compounds might form, a mixture containing Hg^2+^, Zn^2+^, Cd^2+^, Pb^2+^, and Cu^2+^ was analyzed simultaneously in the same solution. Figure S2 illustrates that MIP-PGE provided well-resolved peaks. Despite the presence of multiple heavy metals, including Cu^2+^, which is the closest neighbor in terms of potential, the Hg^2+^ signal remained distinct and highly dominant. The specific binding cavities of MIP facilitated the preferential preconcentration of Hg^2+^, minimizing the suppression often caused by competing ions or intermetallic formation. The imprinting efficiency was assessed by fabricating the nonimprinted NIP-PGE sensor, and the imprinting factor was calculated to imply the effectiveness of the imprinting process.

### 3.5 Imprinting Efficiency of Modified Electrodes

A modified polymer was used to introduce unique binding sites for the analyte on a pencil graphite electrode, enhancing the adsorption onto the electrode surface and improving the specificity. Imprinting efficiency studies were conducted for MIP-PGE and NIP-PGE for a 100 μM concentration of Hg^2+^, as shown in Figure 8. The exact measurement was performed using bare PGE. Using a bare PGE electrode in 100 μM Hg^2+^, no response was obtained. The imprinting factor (I.F.: 16.9) was estimated by dividing the response of MIP-PGE by the response of NIP-PGE. It was determined that the MIP-PGE electrode was 16.9 times more effective than the NIP-PGE electrode for Hg^2+^. This indicates that the formed cavities of MIP-PGE successfully recognize Hg^2+^ in a solution. While the imprinting factor was calculated as 16.9 at a high concentration (100 μM), determining a precise numerical imprinting factor for the lower concentration range (25–150 nM) presented a challenge due to the inherent characteristics of the nonimprinted polymer. In this trace-level range, the NIP-PGE exhibited negligible and often unreliable current responses, indistinguishable from background noise, due to the absence of specific high-affinity binding sites. Consequently, a direct mathematical calculation of the imprinting factor was not feasible for this range. However, this stark contrast, where the MIP-PGE maintains excellent linearity and sensitivity, while the NIP-PGE fails to generate a quantifiable signal, provides compelling qualitative evidence that the specific recognition cavities are the obligatory and dominant mechanism enabling the detection of Hg^2+^ at nanomolar levels.

**Figure 8:**
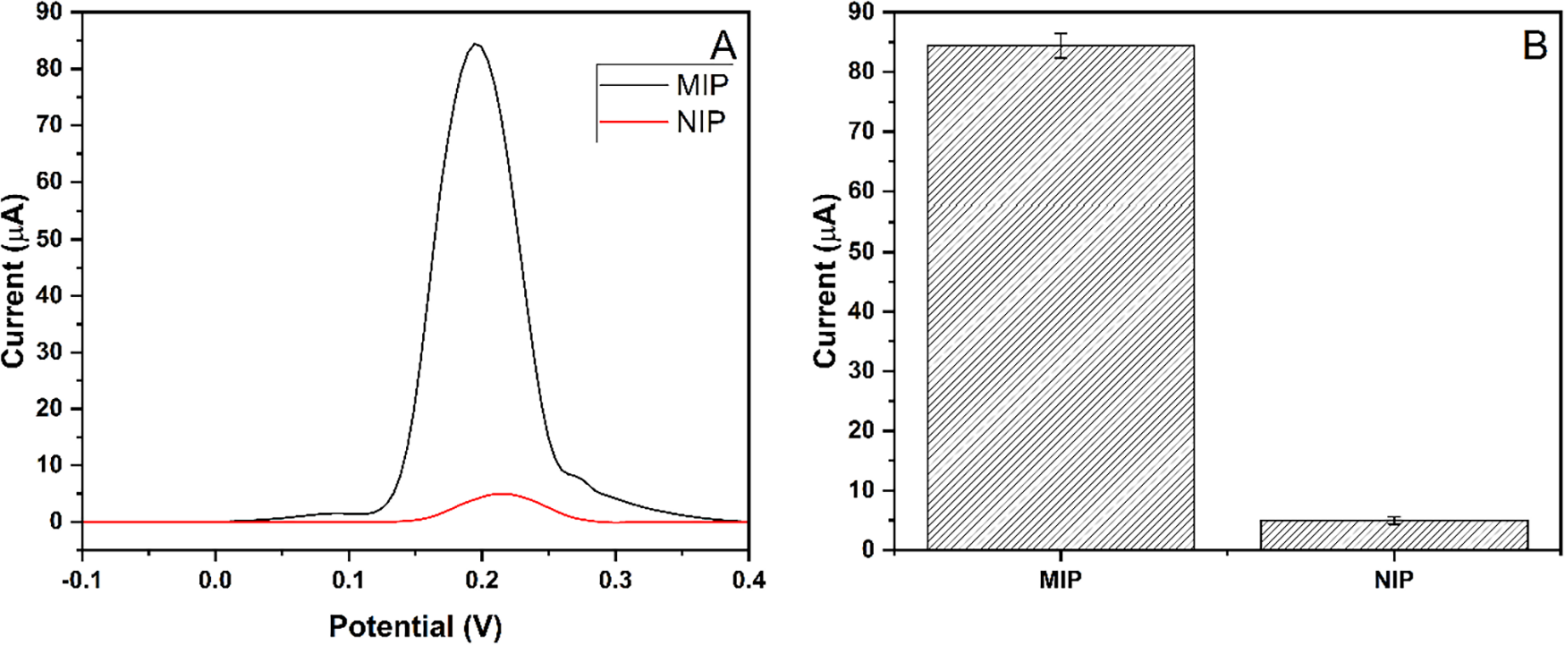
Response of modified electrodes. (A) Voltammogram of MIP-PGE and NIP-PGE responses for 100 μM Hg^2+^ ions. (B) Column representation of the MIP-PGE and NIP-PGE responses with error bars.

### 3.6 Repeatability, Reproducibility, and Stability of MIP-PGE

The repeatability, reproducibility, and stability parameters reported in this section were evaluated by considering the higher calibration range (micromolar level), where the sensor exhibits its primary linear response and saturation behavior. Repeatability and stability of the MIP-PGE were examined by using a 100 μM concentration, and reproducibility was measured using a 50 μM concentration of Hg^2+^ solution. The repeatability was tested using the same solution and MIP-PGE over 10 consecutive measurements (Figure 9), and the relative standard deviation (RSD) was calculated as 1.82%. Based on the slope of the calibration curve, this variation corresponds to an estimated standard deviation of concentration (SD_conc_) of ± 1.81 μM. The percentage difference between the first and last measurements was 4.55%, which represents good repeatability. For the reproducibility test, five MIP-PGEs that have the same monomer-to-template ratio (MIP-B, 1:1) were used, and responses were found as 34.16, 33.86, 34.45, 35.02, and 34.89 μA. The RSD for the response between electrodes for reproducibility was 1.41%, which corresponds to an SD_conc_ of ± 1.34 μM. The slightly higher RSD for repeatability (using the same electrode) compared to reproducibility (different electrodes) can be attributed to the regeneration steps (washing/desorbing with EDTA) required between consecutive measurements on the single electrode. These physical handling and chemical cleaning steps may introduce minor surface variations. In contrast, the reproducibility between different electrodes is remarkably high (1.41%) because the polymerization process used for fabrication is highly automated, controlled, and uniform, resulting in excellent batch-to-batch consistency. The stability of the MIP-PGE was investigated for 20 days (days 1, 2, 3, 4, 5, 10, 15, and 20), as represented in Figure 10. The RSD for 20 days and the percentage difference of the first (day 1) and last (day 20) measurements were 2.19 and 5.69%, respectively. The MIP-PGE retained 94.31% of its initial current response over 20 days.

**Figure 9:**
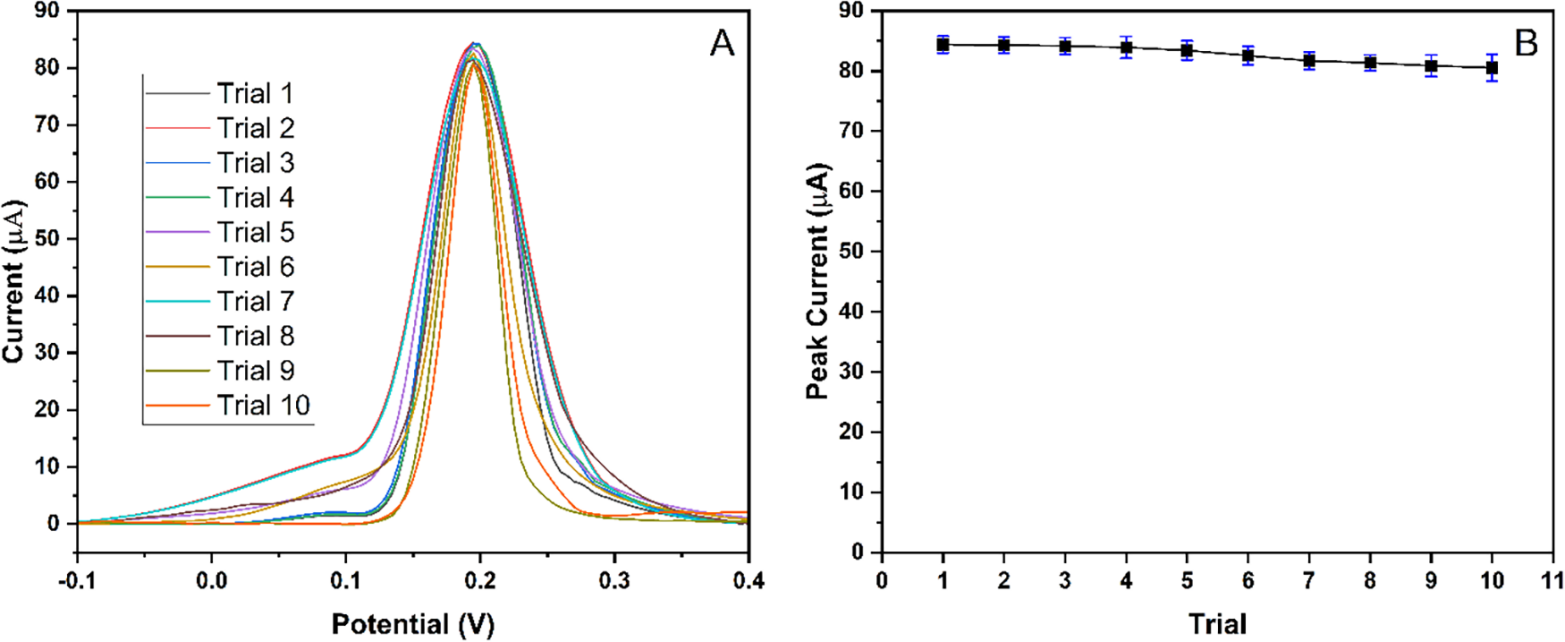
Repeatability study of MIP-PGE for 10 consecutive trials. (A) DPASV responses of MIP-PGE for 100 μM Hg^2+^. (B) DPASV peak current for each trial.

**Figure 10:**
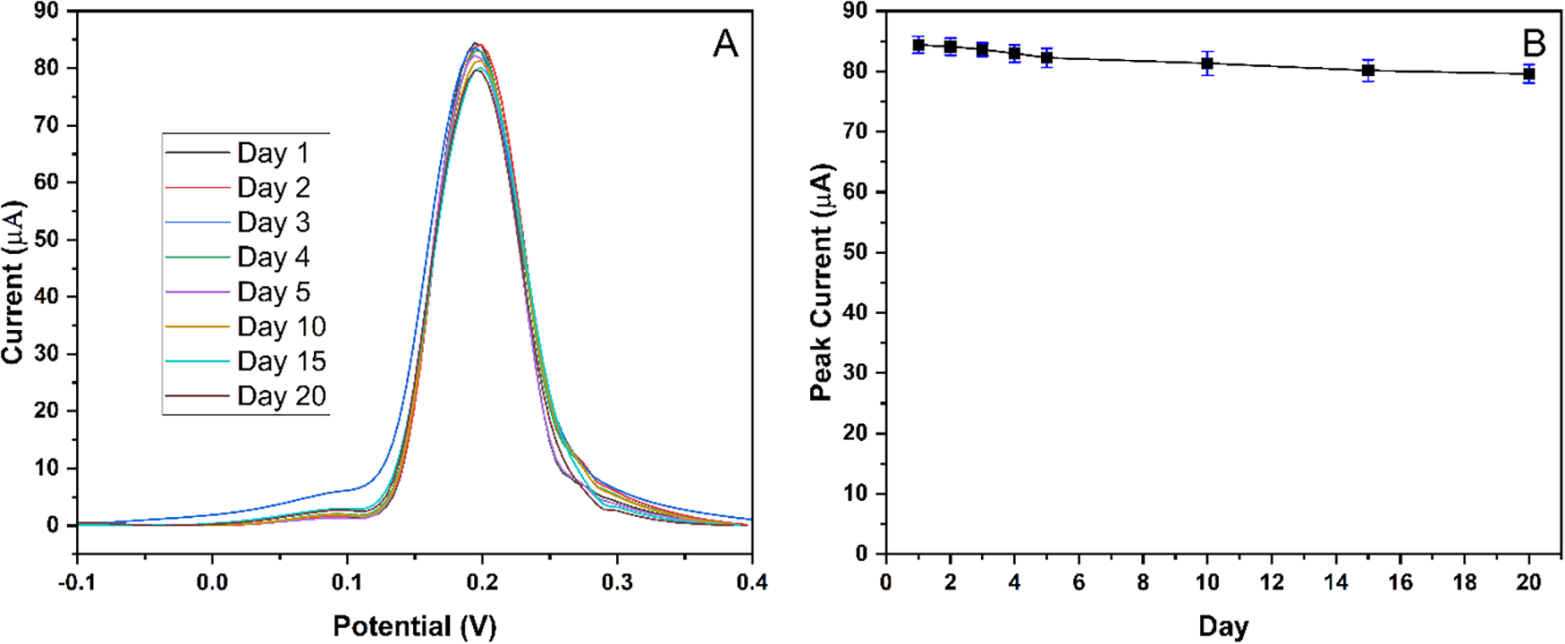
Stability study of MIP-PGE for 20 days. (A) DPASV responses of MIP-PGE for 100 μM Hg^2+^. (B) DPASV peak current for each day.

### 3.7 Determination of Hg^2+^ Ions in a Real Sample

Artificial blood plasma was used as a real sample to determine the detection ability of MIP-PGE in a complex matrix. The determination of Hg^2+^ in artificial blood plasma samples was analyzed by DPASV. For this purpose, 25 to 150 nM Hg^2+^ concentrations were reached by spiking Hg^2+^ in artificial blood plasma. No response to Hg^2+^ was observed when artificial blood plasma was analyzed without the spiking of any Hg^2+^. This result showed that there was no Hg^2+^ in the artificial blood plasma that was used. A similar trend with analytical performance (Figure 6) was observed in the DPASV responses of Hg^2+^ in real samples, as shown in Figure 11A. Figure 11B represents the linear regression equation for Hg^2+^ in artificial blood plasma calibrated as *I =* 0.00656 C + 0.13164 with an *R* ^2^ of 0.9860. These results present an acceptable linear relationship with the peak currents of Hg^2+^ with the increase in the concentration of the target ions. Also, the recovery percentage (%) was calculated to assess the accuracy and reliability of the assay method.^25^ The results indicated that the MIP-PGE electrochemical sensor demonstrated greater accuracy compared to the NIP-PGE sensor. In conclusion, the findings suggest that the MIP-PGE sensor offers an accurate, sensitive, and quantitative assay for measuring the concentration of Hg^2+^ in artificial blood plasma. The recovery of the detected Hg^2+^ molecules was calculated to be higher than 98% for the MIP-PGE electrochemical sensor. These results are summarized in Table 3. Repeatability studies of the MIP-PGE sensor for Hg^2+^ detection were validated with high accuracy and relative standard deviation (RSD < 1.5), demonstrating no significant performance loss. The low RSD of the MIP-PGE sensor demonstrates its high reusability and accuracy.

**Figure 11:**
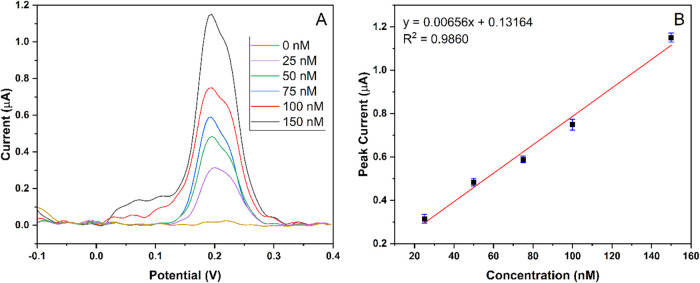
(A) Voltammogram of artificial blood plasma samples containing Hg^2+^ in different concentrations ranging from 25 to 150 nM. (B) Calibration graph of Hg^2+^ in concentrations ranging from 25 to 150 nM.

**Table 3:** Recoveries of Hg^2+^ in Artificial Blood Plasma Samples

	**DPASV**			**CVAAS**		
**added (nM)**	**found (nM)**	**recovery (%)**	**RSD**	**found (nM)**	**recovery (%)**	**RSD**
**25**	25.3	101.2	1.3	24.9	99.6	1.8
**50**	51.1	102.2	1.4	51	98.0	1.1
**75**	74.6	99.4	1.1	74.5	99.3	1.0
**100**	99.4	99.4	1.3	100.7	100.7	1.1
**150**	148.9	99.3	1.5	149.1	99.4	1.4

Cold vapor atomic absorption spectroscopy (CVAAS) at a 254 nm emission spectrum was used to validate the Hg^2+^ detection results recorded by an MIP-PGE sensor. The calibration curve-forming process was performed using the Hg^2+^ standard solutions (25–100 nM, *R* ^2^ > 0.95). Additionally, artificial blood plasma solutions, including Hg^2+^ ions, which were previously applied to the MIP-PGE sensor, were also analyzed by AAS in triplicate. The results provided insight into the efficiency of the MIP-PGE sensor. Additionally, artificial blood plasma solutions, including Hg^2+^ ions, which were previously applied to the MIP-PGE sensor, were also analyzed by AAS in triplicate. The measured Hg^2+^ concentrations were correlated with the corresponding concentrations applied to the MIP-PGE sensor, confirming that the MIP-PGE sensor efficiently detects Hg^2+^ in artificial blood plasma. The calibration curve, constructed using standard Hg^2+^ solutions at concentrations ranging from 25 to 100 nM, exhibited strong linearity (*R* ^2^ = 0.9913, *y* = 0.0195*x* + 0.0384).

### 3.8 Comparison of the MIP-PGE Sensor with the Previously Reported Hg^2+^ Sensors

A comparison of the electrode, detection method, and LOD of the produced MIP-PGE with previously studied voltammetric sensors reported in the literature was made. Various modifications were applied to different electrodes for the detection of Hg^2+^, as listed in Table 4. The LOD of this work is lower than that of some of the studies, such as Yang,^26^ Veerakumar et al.,^27^ Gumpu et al.,^28^ Hao et al.,^29^ and Zhou et al.^30^ On the other hand, some studies used microporous poly­(2-mercaptobenzothiazole)^31^ and tribenzamides^32^ for the modification of electrodes and obtained lower LOD values than this study. However, synthesizing them demands expensive chemicals and includes multistep synthesis reactions, so they require more time. Also, studies that employed square wave anodic stripping voltammetry (SWASV) obtained lower LOD values than those in this study.

**Table 4:** Comparative Performance of Different Voltammetric Sensors for Hg^2+^ Detection

**electrode**	**electrolyte**	**detection method**	**linear range**	***R*** ^**2**^	**LOD** ^a^	**refs**
RGO-IIP/GCE^b^	0.1 M ABS^c^ (pH 4.5)	SWASV^d^	0.350–400 nM	0.999	0.0997 nM	^14^
Hg(II)-imprinted MPMBT/GCE^e^	0.01 M HNO_3_ and 1.0 M KCl	SWASV	1–160 nM	0.987	0.1 nM	^31^
TBa/Ag NPs/GCE^f^	0.1 M HCl (pH 2)	SWASV	0.005–100 nM	0.99	1.7 × 10^–6^ nM	^32^
TAB/CPE^g^	NH_3_- NH_4_Cl buffer (pH 9.64)	anodic dissolution voltammetry	10–100,000 nM	NA (not available)	8.8 nM	^33^
Pd@Bi_2_S_3_/GCE^h^	0.1 M PB (pH 5)	DPV^i^ and LSV^j^	0.49–2475 μM and 0.049–445 μM, respectively	0.9929 and 0.9926, respectively	41.85 and 13.5 nM, respectively	^27^
[Ru(bpy)_3_]^2+^-GO-modified AuE^k^	0.1 M citrate buffer (pH 6)	DPV	100–1200 nM	0.96	1.6 nM	^28^
MIPs/TDNS/AuNPs/MWCNTs/GCE^l^	5 mM K_3_[Fe(CN)_6_]^3–^/^4–^ (containing 0.1 M KCl)	DPV	500–9970 nM	0.994	31.9 nM	^29^
Au–TiO_2_ NPs/chit/gold electrode^m^	0.1 M ABS (pH 5)	DPASV	5–400 nM	0.999	1 nM	^30^
MIP-PGE	0.5 M ABS (pH 4.5)	DPASV	25–150 nM	0.9904	0.188 nM	this study

a Limit of detection.

b Reduced graphene oxide on an ion-imprinted polymer-modified glassy carbon electrode.

c Acetate buffer solution.

d Square wave anodic stripping voltammetry.

e Microporous poly­(2-mercaptobenzothiazole) on a glassy carbon electrode.

f Glassy carbon electrode modified with *N*-{4-[2-(1,3-Benzoxazolyl)]­phenyl}-3,5-*N*,*N*′-bis­(4-octyloxybenzoyl)­benzamide and silver nanoparticles.

g Nitric acid treated acetylene black modified carbon paste electrode

h Nanocomposite containing palladium nanoparticles embedded on bismuth sulfide nanorods on a glassy carbon electrode.

i Differential pulse anodic stripping voltammetry.

j Linear sweep voltammetry.

k Graphene oxide textured ruthenium­(II) bipyridine complex on a gold electrode.

l Glassy carbon electrode modified with chitosan/aptamer (embedded in DNA tetrahedrons) molecularly imprinted polymers, gold nanoparticles, and multiwalled carbon nanotubes.

m Gold electrode modified with gold–titanium dioxide nanoparticles and chitosan.

## 4 Conclusion

This study successfully developed and characterized a novel electrochemical sensor for the highly sensitive and selective detection of Hg^2+^ ions using a pencil graphite electrode modified with a molecularly imprinted polymer (MIP-PGE). The sensor demonstrated excellent analytical performance under optimized conditions, with a low LOD of 0.188 nM and a LOQ of 0.570 nM. The imprinting process proved highly effective, as evidenced by an imprinting factor of 16.9, showing that the MIP-PGE was significantly more effective than its nonimprinted counterpart (NIP-PGE). The sensor also exhibited a high degree of selectivity for Hg^2+^ over competing ions like Cu^2+^, Pb^2+^, Cd^2+^, and Zn^2+^, with selectivity coefficients of 7.62, 3.43, 27.95, and 17.92, respectively. Furthermore, the sensor’s ability to detect Hg^2+^ in a complex matrix was confirmed using artificial blood plasma, yielding similar analytical performance.

This method presents a promising tool for efficient mercury monitoring in environmental and analytical applications. The use of IIPs provides specific recognition sites for Hg^2+^ ions, enhancing selectivity even in complex matrices. The use of PGEs offers a low-cost platform, while voltammetric techniques ensure rapid and sensitive analysis. Briefly, the selective Hg^2+^ determination by MIP-PGE, based on a voltammetric detection method, demonstrated high sensitivity and a low detection limit of 0.188 nM, achieved in just 1 min by the molecular imprinting technique. This process did not require additional steps, such as using a spacer arm or complex processes for ligand immobilization. Also, the selective detection of Hg^2+^ by the molecular imprinting method enables the elimination of the interference effect of other similar ions (Cu^2+^, Pb^2+^, Cd^2+^, and Zn^2+^). By the molecular imprinting technique, template-shaped cavities were created in polymer matrices with predetermined selectivity and high affinity for Hg^2+^ detection.

For future work, this promising platform could be explored for the simultaneous detection of multiple heavy metal ions by designing a multiimprinted polymer with recognition sites for different target analytes. Additionally, integrating this sensor with portable, miniaturized devices could enable on-site, real-time monitoring of mercury in various environmental and biological samples. The core methodology, combining the selectivity of ion-imprinted polymers with the simplicity and cost-effectiveness of PGEs, paves the way for the development of a new generation of electrochemical sensors.

## Supplementary Material



## Notes

Response of MIP-PGE in various micromolar concentrations. (A) Voltammogram of Hg^2+^ by DPASV via the MIP-PGE electrode and (B) calibration graph of Hg^2+^ in concentrations ranging from 0.2 to 0.75 μM (Figure S1). MIP-PGE response to the mixture of Hg^2+^ (100 μM), Cu^2+^ (100 μM), Pb^2+^ (100 μM), Cd^2+^ (100 μM), and Zn^2+^ (100 μM) competitors in the same solution (Figure S2) (PDF)
