# Supplementary material for: Development of a Molecularly Imprinted Pencil Graphite Electrode for the Voltammetric Detection of Hg2+ Ions
Source: ACS Omega. 2026 Jan 23;11(5):7245–56. doi: 10.1021/acsomega.5c07433 (PMC12903017; doi:10.1021/acsomega.5c07433)
Supplement: Supplementary file 1 [file ao5c07433_si_001.pdf]

SUPPLEMENTARY

Development of Molecularly Imprinted Pencil Graphite Electrode for Voltammetric Detection of Hg^{2+} Ions

Mehmet Karagözlü[1,2], Dina El Miari[3], Mariam Moghazi[4], Süleyman Aşır[2,4], Ilgım Göktürk[5], Fatma Yılmaz[6], Adil Denizli[5] and Deniz Türkmen[5*]

[1]*Research Center of Science, Technology and Engineering (BILTEM), Near East University, Mersin 10 Turkey*

[2]*Department of Food Engineering, Faculty of Agriculture, Near East University, Mersin 10 Turkey*

[3]*Department of Biomedical Engineering, Faculty of Engineering, University of Kragujevac, Serbia*

[4]*Department of Biomedical Engineering, Faculty of Engineering, Near East University, Mersin 10 Turkey*

[5]*Department of Chemistry, Hacettepe University, Ankara,06800, Turkey*

[6]*Department of Chemistry and Chemical Processing Technologies, Bolu Abant Izzet Baysal University, Bolu 14030, Turkey*

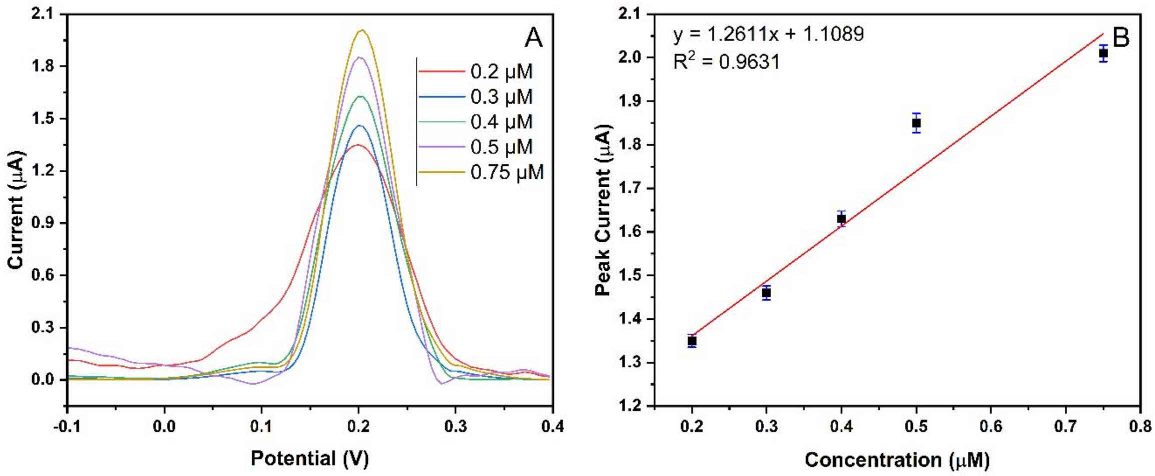

Figure S1. Response of MIP-PGE in various μM concentrations. A) Voltammogram of Hg^{2+} by DPASV via MIP-PGE electrode; B) calibration graph of Hg^{2+} in concentrations ranging from 0.2 μM to 0.75 μM.

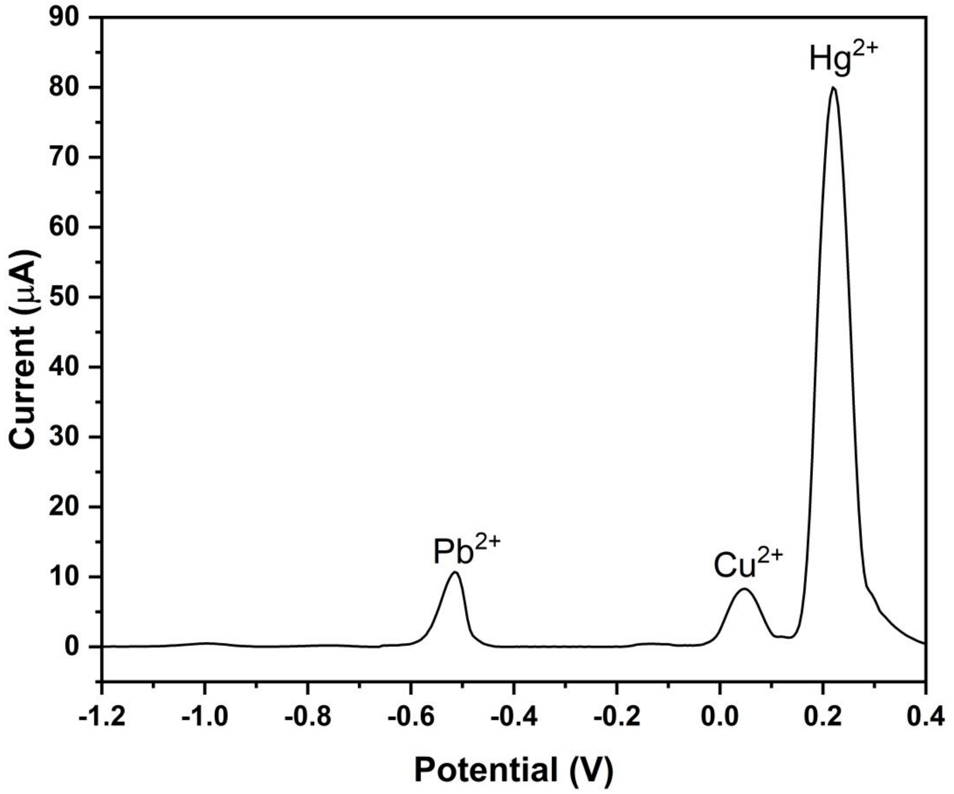

Figure S2. MIP-PGE response to the mixture of Hg^{2+} (100 μM), Cu^{2+} (100 μM), Pb^{2+} (100 μM), Cd^{2+} (100 μM), and Zn^{2+} (100 μM) competitors in the same solution.
